# The Relationship Between Fetal Growth and Retinal Nerve Fiber Layer Thickness in a Cohort of Young Adults

**DOI:** 10.1167/tvst.11.7.8

**Published:** 2022-07-12

**Authors:** Kathleen I. C. Dyer, Paul G. Sanfilippo, Seyhan Yazar, Jamie E. Craig, Alex W. Hewitt, John P. Newnham, David A. Mackey, Samantha S. Y. Lee

**Affiliations:** 1 Centre for Ophthalmology and Visual Sciences (incorporating the Lions Eye Institute), University of Western Australia, Nedlands, Australia; 2 Centre for Eye Research Australia, Royal Victorian Eye and Ear Hospital, University of Melbourne, Melbourne, Australia; 3 Single Cell and Computational Genomics Laboratory, Garvan Institute of Medical Research, Darlinghurst, Australia; 4 Eye and Vision, Flinders Health and Medical Institute, Flinders University, Adelaide, Australia; 5 School of Medicine, Menzies Research Institute Tasmania, University of Tasmania, Hobart, Australia; 6 School of Women's and Infants’ Health, The University of Western Australia, Perth, Australia

**Keywords:** retinal nerve fiber layer thickness, optic nerve, fetal growth, fetal head circumference, Raine Study

## Abstract

**Purpose:**

To explore relationships between patterns of fetal anthropometric growth, as reflective of fetal wellbeing, and global retinal nerve fiber layer (RNFL) thickness measured in young adulthood.

**Methods:**

Participants (*n* = 481) from within a Western Australian pregnancy cohort study underwent five serial ultrasound scans during gestation, with fetal biometry measured at each scan. Optic disc parameters were measured via spectral-domain optical coherence tomography imaging at a 20-year follow-up eye examination. Generalized estimating equations were used to evaluate differences in global RNFL thickness between groups of participants who had undergone similar growth trajectories based on fetal head circumference (FHC), abdominal circumference (FAC), femur length (FFL), and estimated fetal weight (EFW).

**Results:**

Participants with consistently large FHCs throughout gestation had significantly thicker global RNFLs than those with any other pattern of FHC growth (*P* = 0.023), even after adjustment for potential confounders (*P* = 0.037). Based on model fit statistics, FHC growth trajectory was a better predictor of global RNFL thickness than birth weight or head circumference at birth. RNFL thickness did not vary significantly between groups of participants with different growth trajectories based on FAC, FFL, or EFW.

**Conclusions:**

FHC growth is associated with RNFL thickness in young adulthood and, moreover, is a better predictor than either birth weight or head circumference at birth.

**Translational Relevance:**

This research demonstrates an association between intrauterine growth and long-term optic nerve health, providing a basis for further exploring the extent of the influence of fetal wellbeing on clinical conditions linked to RNFL thinning.

## Introduction

The optic nerve develops during fetal life and during early childhood. As early as 4 weeks’ gestation, it is possible to distinguish the optic stalk, which eventually forms the optic nerve.^1^ In full-term neonates, approximately 75% of optic nerve development is complete by birth and 95% by 1 year of age.^2^^,^^3^ Current evidence suggests that this process can be altered by exposures present during gestation such as maternal smoking, which has been identified as an independent risk factor for decreased retinal nerve fiber layer (RNFL) thickness by early adolescence and young adulthood.^4^^–^^6^ Moreover, several studies have demonstrated that deviations in birth parameters such as low birth weight and small head circumference, which may reflect adverse early life conditions and fetal growth restriction, are associated with changes in optic nerve morphology, including thinner RNFLs, decreased neuroretinal rim areas, smaller optic discs, and larger cup-to-disc ratios measured during childhood.^3^^,^^4^^,^^7^^–^^10^ There is emerging evidence that these associations persist into adulthood, with a recent analysis from the population-based Gutenberg Health Study demonstrating a significant relationship between self-reported birth weight and peripapillary RNFL thickness in a cohort of 35- to 74-year-old individuals.^2^

Birth weight and head circumference at birth are easily and routinely obtained measures and thus are frequently used as surrogate markers of fetal growth. However, such measures are often unreliable, particularly as they provide limited insight into intrauterine growth rate and changes in this growth rate throughout gestation. Indeed, not all neonates with low birth weight or small head circumference will have necessarily undergone fetal growth restriction or will experience any of the associated complications.^11^^–^^14^ Conversely, it has been estimated that up to 47% of neonates diagnosed with intrauterine growth restriction based on fetal anthropometry measured via serial ultrasound scans are born at a normal weight for gestational age.^15^

Existing research into the relationship between RNFL thickness and early life development has largely relied on single birth parameter measures as surrogates of fetal growth and wellbeing, with no published studies having used longitudinal measurements performed during gestation. Therefore, it is currently unknown whether the relationships between neonatal anthropometry and RNFL thickness later in life established thus far do indeed reflect the influence of fetal growth and wellbeing or simply result from size proportion. RNFL thinning is characteristic of a number of optic and retinal neuropathies and, in particular, is one of the main diagnostic markers of glaucoma.^16^^–^^18^ It is often accompanied by other changes in measures of retinal ganglion cell integrity, including thinning of the macular ganglion cell–inner plexiform layer (GCIPL) and an increase in the optic cup-to-disc ratio.^19^ Furthermore, evidence suggests that there may be an association between the risk of developing glaucoma and premorbid optic disc measures.^20^ A better understanding of factors that influence baseline optic nerve morphology may therefore enable the development of strategies for timely detection of conditions such as glaucoma. This is clinically relevant, given that glaucoma is often diagnosed only after visual changes become noticeable, by which stage significant optic nerve damage may have already occurred.

In this longitudinal cohort study, we examined possible long-term effects of fetal wellbeing on optic nerve development by exploring associations between intrauterine growth trajectories based on serial ultrasound measurements of fetal head circumference (FHC), abdominal circumference (FAC), femur length (FFL), and estimated fetal weight (EFW), and RNFL thickness in young adulthood. Additional markers of optic nerve health, vertical cup-to-disc ratio (VCDR) and GCIPL thickness, were examined with respect to growth trajectories in secondary analyses.

## Methods

### Study Design

This analysis was performed using data from the Raine Study, a multigenerational cohort study based in Perth, Western Australia. At 16 to 18 weeks’ gestation, 2900 pregnant women from the King Edward Memorial Hospital antenatal clinic and nearby private clinics were enrolled in the Raine Study between 1989 and 1991 (Gen1 participants). The original study design incorporated an investigation of the effects of frequent ultrasound scans during pregnancy on birth outcomes. Half of the Gen1 participants were randomly allocated to “intensive care”, with ultrasound imaging performed at 18, 24, 28, 34, and 38 weeks’ gestation; the other half were assigned to “regular care”, involving a single ultrasound scan at 18 weeks’ gestation only, unless subsequent scans were clinically indicated.^21^ These women's offspring (Gen2 participants) have since formed a prospective cohort study, undergoing a series of follow-up assessments relating to a wide range of health outcomes. All active Gen2 participants were invited to the Gen2-20 year follow-up, when the cohort underwent a comprehensive ophthalmic examination for the first time.

Upon enrollment, all Gen1 participants provided written informed consent. The ethics committees at King Edward Memorial Hospital, Princess Margaret Hospital, and the University of Western Australia approved the protocol for data collection during gestation. For the Gen2-20 year follow-up, Gen2 participants provided their own informed consent, and the protocol for ophthalmic data collection was approved by the Human Research Ethics Committee at the University of Western Australia. Protocols for data collection during gestation and at all Raine Study follow-up assessments adhered to the tenets of the Declaration of Helsinki. The Raine Study is registered in the Australian New Zealand Clinical Trials Registry.^22^

### Study Participants

**Figure 1.**
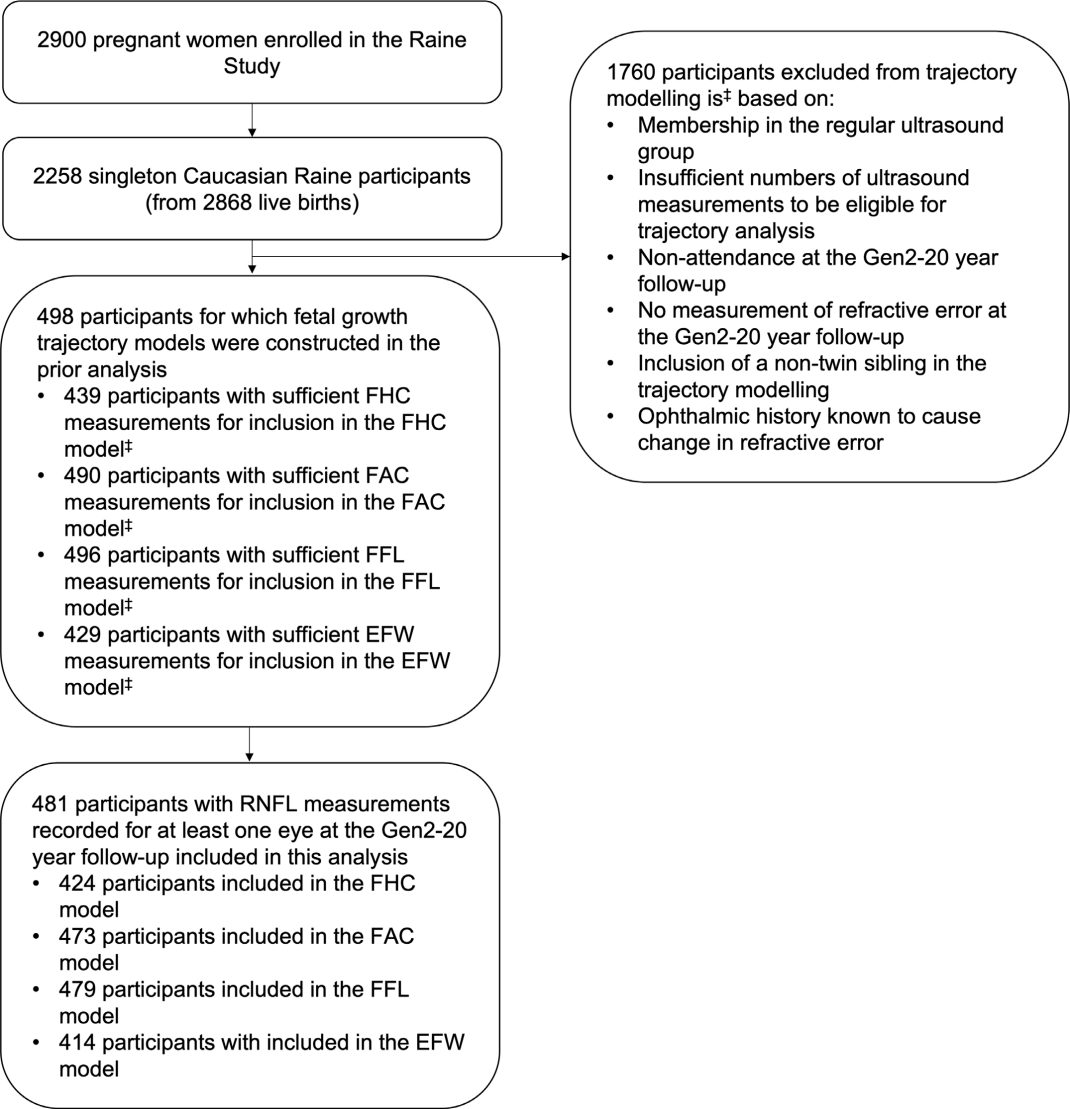
Study population flow chart. ^‡^A breakdown of the study population used for trajectory modeling is described in detail in the previous study by Dyer et al.^23^

This analysis examined RNFL thickness at 20 years of age with respect to fetal growth trajectory models based on FHC, FAC, FFL, and EFW that we previously constructed in a 2020 investigation into the relationships between fetal growth trajectories and the ocular outcomes of myopia, axial length and corneal radius of curvature within the Raine Study.^23^ The models had been constructed for a subset of 498 Gen2 participants from the “intensive care” group, for whom at least one fetal biometric parameter had been measured around at least four of the five nominal time points in order to enable modeling of growth trajectory and for whom refractive error measurements had been recorded at the Gen2-20 year follow-up. Of these participants, 481 had RNFL thickness measurements recorded for at least one eye at the Gen2-20 year follow-up and were therefore included in the current study, as shown in Figure 1.

A number of additional criteria used for participant selection in the 2020 study in which the trajectory models were constructed^23^ also applied to the current analysis. All included participants were of Caucasian ethnicity, as only a small proportion of Gen2 participants were of non-Caucasian or mixed ethnicities (14.5%), limiting the statistical ability to examine the confounding effects of this variable. Participants from multiple pregnancies (twins and triplets) were also excluded given the known association with slower fetal growth, increased rates of preterm birth, and lower birth weights.^24^ In cases where multiple non-twin siblings were eligible for inclusion in the analysis, one sibling only was randomly chosen to be included in order to reduce bias due to genetic similarity. The specific numbers of participants excluded based on the above criteria have been detailed previously.^23^

### Fetal and Neonatal Examinations

Intrauterine imaging was performed by qualified sonographers as previously described^21^^,^^25^ using one of two General Electric RT 3600 machines (Milwaukee, WI). Parameters measured included FHC, FAC, and FFL. Each parameter was measured three times to the nearest millimeter, and the three measurements were averaged for use in the analysis. An EFW for each visit was derived from the FHC, FAC, and FFL measurements according to a Hadlock formula.^26^ Neonatal biometry was performed by extensively trained midwives. Birth weight was measured to the nearest 100 g using calibrated hospital scales, and head circumference at birth was measured using a precise paper tape to the nearest millimeter. Gen1 participants completed questionnaires pertaining to baseline maternal characteristics at 18 and 34 weeks’ gestation, including information about cigarette smoking during the pregnancy. In this analysis, Gen2 participants were considered to have had “significant” exposure to maternal smoking during pregnancy if their mother reported being a current smoker at 18 weeks’ gestation, and “limited” exposure otherwise.

### Fetal Growth Trajectories

The methods for the construction of the FHC, FAC, FFL, and EFW trajectory models have previously been described in detail in our prior study.^23^ In brief, each fetal growth parameter measurement was converted to a population-based standard deviation score for gestational age using linear mixed-effects modeling with adjustments for maternal height, maternal age, and fetal sex, in R 4.0.4 (R Foundation for Statistical Computing, Vienna, Austria). Clusters of participants undergoing similar growth trajectories based on these standard deviation scores were then determined using a group-based trajectory modeling approach developed by Nagin.^27^ Group-based trajectory modeling is designed to identify latent subgroups within a population according to developmental trajectories based on a longitudinally measured variable. The use of this technique is becoming increasingly widespread across multiple fields of healthcare research, including maternal–fetal and pediatric medicine and the visual sciences.^28^^–^^33^ In this analysis, group-based trajectory modeling was performed using the Proc Traj add-on package within SAS 9.4 (SAS Institute, Inc., Cary, NC).^34^

Supplementary Figure S1 displays plots of the four trajectory models constructed in our previous study, which were used in this analysis.^23^ The FHC and FFL trajectory models both consisted of five groups, four of which reflected participants with relatively stable growth throughout gestation (labeled “small”, “medium”, “big”, and “large”) and one group of participants whose growth rate increased throughout gestation relative to the rest of the study population (labeled “accelerated”). There were only four groups in the FAC trajectory model, which included an “accelerated” trajectory group in addition to three stable trajectory groups (“small”, “medium” and “large”). The EFW model consisted of six groups, including one “small” and one “large” stable trajectory groups, and four groups of medium-sized participants, each showing moderate amounts of either deceleration (the “medium-small” and “big-medium” groups) or acceleration (the “medium-big” and “big-large” groups).

### Ophthalmic Examination

The Gen2-20 year follow-up, which included a comprehensive ophthalmic examination, was completed between 2010 and 2012. As outlined by Yazar et al.,^35^ the examination included ocular biometry (IOLMaster 5; Carl Zeiss Meditec, Jena, Germany), tonometry (TAO1i Rebound Tonometer; iCare Finland Oy, Vantaa, Finland), and spectral-domain optical coherence tomography (SD-OCT) imaging of the optic disc (SPECTRALIS HRA+OCT; Heidelberg Engineering, Heidelberg, Germany). All SD-OCT imaging was performed by trained technicians as described previously,^36^ and the signal-to-noise ratios were maintained above 20 during scans.^37^ Disc measures obtained included global and sectoral peripapillary RNFL thicknesses, optic cup and Bruch's membrane opening (BMO) vertical and horizontal widths, and GCIPL thicknesses. Supplementary Figure S2 shows the six sectors defined for RNFL thickness, as well as the regions in which GCIPL thicknesses were described, which included eight of the nine regions defined according to the Early Treatment Diabetic Retinopathy Study (ETDRS).^38^ The central ETDRS region was excluded because the GCIPL is absent in the central macula. VCDRs were calculated based on the BMO and optic cup widths as previously described,^39^ as the BMO provides a better indication of the anatomical border of the neuroretinal rim than the visible disc margin.^40^ Scans were reviewed offline, and those with segmentation errors were corrected. All analyses involving the VCDR were corrected for the vertical BMO width.

### Statistical Analyses

Statistical analyses for this study were conducted using R 3.6.3. Differences in global RNFL thickness between trajectory groups were examined using generalized estimating equations (GEEs). We chose to use GEEs to account for missing data, the non-normal distribution of the data, and adjustments for covariates. Additionally, the exchangeable correlation structure of the GEE models allowed for both eyes of each participant to be included, thus increasing statistical power while accounting for the within-subject correlation between the two eyes.^41^^,^^42^ The GEE models were adjusted for the potential confounders of gestational age at birth, exposure to maternal smoking during pregnancy, and intraocular pressure and axial length measured at the Gen2-20 year follow-up, in view of the known influences of these variables on RNFL thickness or the development of glaucoma.^6^^,^^7^^,^^43^^–^^50^

Where trajectory group membership in a fetal growth trajectory model was determined to be significantly associated with global RNFL thickness, we also assessed which of fetal growth trajectory group membership, birth weight, or head circumference at birth, was the best predictor of global RNFL thickness. This was done by reconstructing the GEE model with trajectory group membership replaced first by birth weight, then head circumference at birth, as the independent variable, with adjustments made for the covariates previously mentioned. The quasi-likelihood information criterion (QIC) was then used to compare the three GEE models, with a lower QIC indicating better model fit.^51^ Fetal growth trajectory models that showed a significant association between group membership and global RNFL thickness were further examined with respect to sectoral RNFL thicknesses and secondary ophthalmic outcomes including VCDR and GCIPL thickness, using GEE models in the same manner as described above for the global RNFL thickness analyses.

## Results

### Descriptive Analysis

Of the 481 participants included in this analysis, 239 were male (49.7%), and the mean age ± SD was 20.1 ± 0.4 years at the time of the ophthalmic examination. There were no significant differences in the distributions of sex or ethnicity between participants who attended the Gen2-20 year follow-up and were excluded from the analysis and those who were included (*P* > 0.05). Trajectory data for FHC, FAC, FFL, and EFW were available for 424, 473, 479, and 414 participants in the study population, respectively. The distributions of a variety of maternal, pregnancy and neonatal characteristics across the trajectory groups in these models have previously been published.^23^ Supplementary Table S1 contains the distributions of several specific variables known to be associated with RNFL thickness in adult life—namely, fetal sex, exposure to maternal smoking during pregnancy, gestational age at birth, birth weight, and head circumference at birth, as well as intraocular pressure and axial length measured at the Gen2-20 year follow-up. In all four trajectory models, distributions of fetal sex and gestational age at birth were relatively similar across the trajectory groups. Trajectory groups representing smaller fetal size or slower fetal growth during gestation consistently showed a higher prevalence of significant exposure to maternal smoking during pregnancy, as well as lower birth weights and head circumferences at birth in all of the models. Meanwhile, consistent with results from our previous study,^23^ there was a trend toward increased axial length at the Gen2-20 year follow-up among groups representing faster fetal growth according to the FHC, FFL, and EFW trajectory models, although not at a statistically significant level. There were no significant differences in intraocular pressure between the trajectory groups in any model.

### RNFL Thickness Across Fetal Growth Trajectories

**Figure 2.**
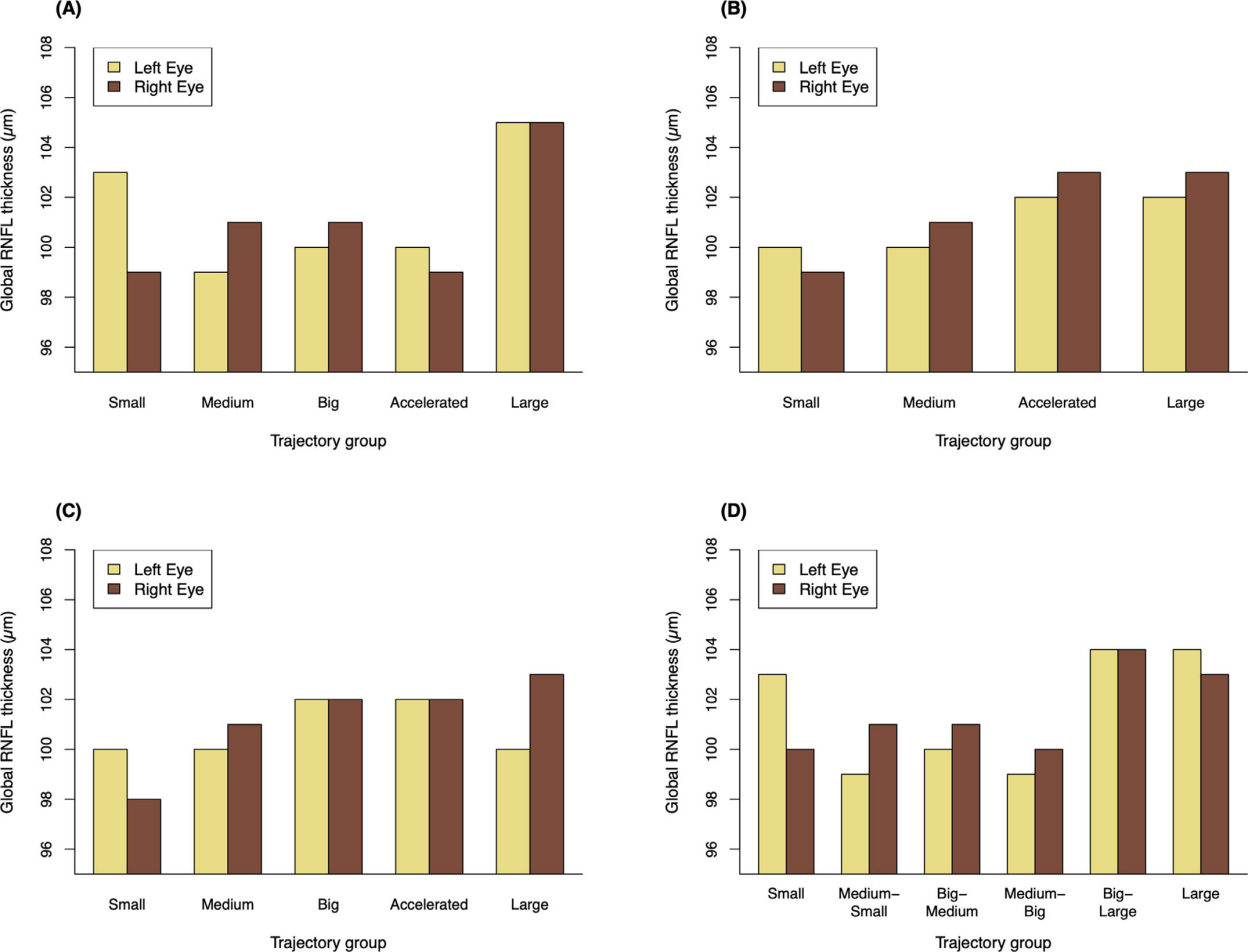
Median global RNFL thicknesses for the trajectory groups of each model. (**A**) FHC model; (**B**) FAC model; (**C**) FFL model; and (**D**) EFW model. Each panel presents the median left and right global RNFL thicknesses for each model group. In the FHC trajectory model, *P* < 0.05 when calculated using generalized estimating equations both with and without adjustment for the potential confounders of gestational age at birth, maternal smoking during pregnancy, and axial length and intraocular pressure measured at the Gen2-20 year follow-up. In each of the other trajectory models, *P* > 0.05 for both unadjusted and adjusted analyses.

The median global RNFL thickness across the 481 study participants was 101 µm (interquartile range [IQR], 94–107) in the right eye and 101 µm (IQR, 95–107) in the left eye. The distributions of global RNFL thickness across trajectory model groups are presented in Figure 2 and Supplementary Table S2. Global RNFL thickness was associated with trajectory group membership in the FHC model at a statistically significant level (*P* = 0.023), even after adjusting for potential confounders (*P* = 0.037). In particular, participants in the “large” FHC trajectory group had considerably thicker global RNFLs than the other four groups, with median global RNFL thicknesses of 105 µm (IQR, 102–110) in the right eye and 105 µm (IQR, 98–109) in the left eye. We assessed the differences in global RNFL thickness between the “large” group and the other groups more closely by examining the trajectory group coefficient data from the FHC GEE models. As demonstrated in the Table, differences between the “large” group and each of the other four groups were statistically significant in both unadjusted and adjusted analyses, taking into account the Bonferroni correction (*P* < 0.05/4 = 0.0125 for four comparisons). There were no significant differences in global RNFL thickness among the other FHC groups (Supplementary Table S3). The FAC, FFL, and EFW GEE models showed trends similar to those for the FHC GEE models; that is, global RNFL thickness was generally greater in groups exhibiting faster fetal growth or larger fetal size throughout gestation. However, as demonstrated in Supplementary Table S2, these trends did not reach statistical significance.

**Table.** Associations Between Global RNFL Thickness and FHC Trajectory Group Membership, Using the “Large” Group as a Reference

	“Large” Group			
	Unadjusted		Adjusted	
Trajectory Group	Estimate (95% CI)	*P* ^*^	Estimate (95% CI)	*P* ^*^
Small	−4.04 (−7.00 to −1.09)	0.007	−4.01 (−7.04 to −0.99)	0.009
Medium	−3.79 (−6.13 to −1.45)	0.002	−3.73 (−6.20 to −1.27)	0.003
Big	−3.70 (−6.04 to −1.36)	0.002	−3.69 (−6.14 to −1.25)	0.003^*^
Accelerated	−3.63 (−6.47 to −0.80)	0.012	−4.07 (−7.00 to −1.13)	0.007

Estimates and *P* values have been calculated in GEEs both in an unadjusted model and in a model adjusted for the covariates of gestational age at birth, exposure to maternal smoking during pregnancy, and intraocular pressure and axial length at the Gen2-20 year follow-up.

* Significant at *P* < 0.0125 (=0.05/4 taking into account Bonferroni correction for four comparisons).

The QIC associated with the FHC GEE model for global RNFL thickness, adjusted for potential confounders, was calculated to be 59822.28. By comparison, higher QICs were determined when the FHC trajectory group variable was replaced with birth weight or head circumference (67481.91 and 66762.68, respectively). This suggests a better model fit for the FHC GEE model and that FHC trajectory is a better predictor of global RNFL than either birth weight or head circumference at birth.

When examined by sector, RNFL thickness was again noticeably greater in the “large” group compared to the other trajectory groups in all except the superonasal sector, as demonstrated in Supplementary Table S4. Taking into account the Bonferroni correction for six analyses, this difference reached statistical significance for the nasal sector in the adjusted analysis only (*P* = 0.002). Supplementary Table S4 also shows the distributions of the secondary ophthalmic outcomes by FHC trajectory group. Although VCDR did not vary appreciably between the groups, GCIPL thickness was generally greatest in the “large” group and lowest in the “small” group across all eight ETDRS regions. Statistically significant associations between GCIPL thickness and FHC trajectory group membership, with a Bonferroni correction for eight analyses, were demonstrated for the outer temporal and superior regions in both the unadjusted analyses (*P* = 0.0001 and *P* = 0.003, respectively) and when adjusted for potential confounders (*P* = 0.0002 and *P* = 0.002, respectively), and for the outer nasal region in the adjusted analysis only (*P* = 0.005).

## Discussion

In this study, we identified a significant association between FHC trajectory and global RNFL thickness at 20 years of age in a cohort of singleton Caucasian participants. This association remained significant when adjustments were made for a number of potential confounders. Specifically, individuals with consistently large FHC throughout gestation had thicker global RNFLs compared to individuals with any other type of FHC growth trajectory.

Our findings strengthen prior evidence for fetal programming of long-term optic nerve health. In 2011, the Sydney Eye Health Study^8^ reported that every centimeter increase in head circumference at birth and every kilogram increase in birth weight were associated with increases of 0.44 µm and 2.97 µm, respectively, in average RNFL thickness at 12 years of age. This positive association between birth weight and RNFL thickness in childhood has since been replicated in the prospective Copenhagen Child Cohort 2000 Eye Study^5^ and the cross-sectional EFFORT (Environmental Fetal Factors in the development of the Optic nerve and the Retina) study.^9^ A recent cross-sectional analysis of 35- to 74-year-old participants from the Gutenberg Health Study^2^ further demonstrated a positive association between birth weight and RNFL thickness that persists into adulthood, both globally and in all except the temporal sector.

Our observations of increased RNFL thicknesses among individuals with faster FHC growth throughout gestation and corresponding trends for those with faster FAC, FFL, and EFW growth are consistent with previously published findings. Furthermore, our research extends on those findings by characterizing fetal growth via trajectory modeling based on measurements made during gestation rather than using cross-sectional birth parameters, such as birth weight or head circumference at birth, that lack reliability as surrogate markers of fetal wellbeing. Moreover, we found that FHC growth trajectory was a better predictor of global RNFL thickness than either of these cross-sectional parameters. This finding helps to validate the hypothesis that the previously demonstrated relationships between RNFL thickness and birth parameters are a reflection of the underlying influence of fetal wellbeing on long-term optic nerve health.

The association between global RNFL thickness and FHC growth trajectory was chiefly driven by the distribution of RNFL thickness in the “large” FHC trajectory group, which was significantly higher than in all the other groups, including the “accelerated” group. This is despite the “large” and “accelerated” groups being comprised of individuals with comparable FHCs toward the end of gestation and very similar distributions of birth head circumference, with the mean birth head circumference of the “accelerated” group measuring slightly higher than that of the “large” group, as seen in Supplementary Table S1. In contrast, the two groups primarily differed from each other with respect to FHC distribution at mid-pregnancy, suggestive of much faster FHC growth in the “large” group prior to 18 weeks’ gestation. Axon proliferation within the optic nerve largely occurs in the first and early second trimesters, with the later part of gestation being characterized by the elimination of a significant number of supernumerary axons and associated optic nerve remodeling.^52^^,^^53^ It is plausible that certain factors stimulating growth during early gestation and resulting in the larger FHCs by mid-pregnancy observed in the “large” group compared with the others would also promote increased axon proliferation and thus predispose toward thicker RNFLs in the longer term. The results of our analysis of GCIPL with respect to FHC growth trajectory also suggest that such a process likely occurs during early pregnancy. In the eighth week of gestation, the inner plexiform layer begins to grow outward from the fovea, reaching the periphery of the retina by approximately the 18th week.^54^ Our analysis found a trend toward greater GCIPL thickness in groups representing faster FHC growth in all ETDRS regions, which parallels the observed relationship between FHC growth trajectory and global RNFL thickness. This trend was most pronounced in the outer regions, suggesting that any conditions that concurrently affect FHC growth and optic nerve development likely take effect while the inner plexiform layer is still forming in the outer retina during early to mid-gestation.

A process that promotes both FHC growth during early gestation and greater RNFL thickness in adulthood could be genetically driven, as previous twin and family studies have identified a high heritability of RNFL thickness,^55^^,^^56^ but it could also reflect the impact of early in utero environmental exposures. Interestingly, when RNFL thickness was analyzed by sector, nasal RNFL thickness was determined to most strongly associate with FHC trajectory group membership. Thinner superior and nasal RNFLs have also previously been identified in preterm compared to full-term infants in a 2015 prospective cohort study by Park and Oh,^57^ who furthermore found a correlation between thinner nasal RNFLs and the development and severity of retinopathy of prematurity in preterm infants. These results point toward a particular vulnerability of the nasal sector to structural alterations due to exposures that disrupt prenatal development, which may overlap with pathological processes predisposing toward preterm birth and retinopathy of prematurity. Associations between exposure to maternal smoking during pregnancy and thinner RNFL in adolescence and young adulthood have previously been demonstrated^4^^–^^6^; however, there are limited data on the effects of other specific gestational conditions on optic nerve development. It can also be noted that our analysis found no clear association between FHC growth trajectory and VCDR, another important marker of optic nerve health. This does not align with previous results from the large Sydney Eye Health Study,^10^ which is the other existing analysis to have investigated the relationship between fetal growth markers and VCDR. That study identified a significant increase in VCDR in 12-year-old children with lower birth weights and head circumferences. Identifying and understanding the exact mechanisms that link optic nerve health and FHC growth will require further, more detailed investigation involving larger population sizes than in the present study.

The trends in global RNFL thickness observed with respect to the FAC, FFL, and EFW trajectory models parallel the association demonstrated in the FHC trajectory model. This most likely reflects the correlation between these three parameters and FHC, rather than a direct influence of the growth rate of these parameters on optic nerve development. Indeed, the majority of the “large” FHC trajectory group members are distributed among groups in the other trajectory models that also represent faster growth during early gestation, with 73.3% belonging to the “large” FAC group, 76.7% to either the “large” or “big” FFL group, and 100% to the “large,” “big-large,” or “big-medium” EFW groups.

The association identified between FHC growth and baseline RNFL thickness at 20 years of age provides a basis for investigating the extent to which fetal wellbeing may contribute to the risk of developing conditions linked to changes in optic nerve morphology. Such conditions include glaucoma, a disease characterized by thinning of the RNFL. Our results may also have implications for differences in neurodevelopment according to various FHC growth patterns, with existing evidence suggesting an association between RNFL thinning and poorer cognitive function,^58^^–^^60^ as well as a number of structural intracranial pathologies.^61^ In a large prospective cohort, the INTERBIO-21st Fetal Study^28^ showed significantly improved neurodevelopmental outcomes by 2 years of age, particularly in visual and language domains, for individuals with FHC growth trajectories characterized by larger FHC by the end of the second trimester, similar to the “large” FHC trajectory group in our analysis. Our finding of thicker RNFLs in these individuals at 20 years of age may therefore reflect the long-term persistence of underlying differences in neural structure that correlate with clinical outcomes assessed in the INTERBIO-21st study. However, it should be noted that the FHC GEE model explained only a small amount of variance in global RNFL thickness according to the *R*^2^ equivalent (0.01 in the unadjusted model), calculated using a formula developed by Zheng.^62^ Thus, the extent to which the observed relationship in this study translates into clinically evident differences remains unclear. As our cohort continues to be followed into adulthood, serial measurements of optic disc parameters and collection of clinical data relating to glaucoma diagnoses and neurological outcomes will allow further elaboration.

A primary strength of this study was our comprehensive dataset, including serial anthropometric measurements obtained during gestation and at birth, allowing for detailed characterization of fetal growth via trajectory modeling. This also enabled comparison of these growth trajectories as predictors of global RNFL thickness with the cross-sectional birth parameters of birth weight and head circumference at birth, which have been used as surrogate markers of fetal growth in previous investigations of the relationship between early-life wellbeing and RNFL thickness in both childhood and adulthood. Another strength is the measurement of follow-up ophthalmic outcomes via high-resolution imaging with SD-OCT across a narrow age range in young adulthood prior to the usual age of diagnosis for glaucoma, which is typically greater than 40 years.^63^ Our data therefore capture preclinical variations in RNFL thickness, with minimal likelihood of bias in the analysis that would be associated with the potential confounding variables of age at follow-up or existing glaucoma diagnoses.

A major limitation was the moderate cohort size of this study, as we were consequently unable to identify groups of individuals with growth rates that appreciably decelerated relative to the rest of the study population and thus assess the effects of such growth patterns on RNFL thickness. Certain trajectory groups, specifically the “small” and “large” groups in the FHC, FFL, and EFW models, were also quite small, with participant numbers as low as 28 in the “small” FHC group, so the results of our analyses should be interpreted conservatively. Furthermore, the findings of our study are limited to Caucasian populations only. Previous literature has determined that both fetal growth trajectories and RNFL thickness vary considerably with ethnicity.^44^^,^^45^^,^^64^^,^^65^ In the Raine Study cohort specifically, a recent publication by Lingham et al.^66^ reported thinner global RNFLs in participants of East and Southeast Asian descent than in Caucasian participants. These differences did not remain significant after adjustment for axial length, although the authors noted that this may have again been in part due to the small numbers of non-Caucasian Raine Study participants. Larger study populations with greater proportions of non-Caucasian participants are therefore required in order to validate our study results and explore their generalizability to different ethnic populations.

In summary, our analysis provides evidence to support the hypothesis that long-term optic nerve health is influenced by fetal wellbeing as quantified by FHC growth trajectories, with larger FHC from early in gestation significantly associating with thicker global RNFL by 20 years of age. These associations likely result from both genetic factors and alterations in environmental conditions during the first and second trimesters that concurrently affect growth of FHC and the optic nerve, which undergoes significant development during this period of gestation. The Raine Study provides an opportunity to explore the extent to which our findings have long-term clinical significance for conditions linked to RNFL thinning, such as glaucoma, as the Gen2 participants continue to be followed through adult life.

## Supplementary Material

Supplement 1

Supplement 2

Supplement 3

Supplement 4

Supplement 5
