# Supplementary material for: The Relationship Between Fetal Growth and Retinal Nerve Fiber Layer Thickness in a Cohort of Young Adults
Source: Transl Vis Sci Technol. 2022 Jul 12;11(7):8. doi: 10.1167/tvst.11.7.8 (PMC9287618; doi:10.1167/tvst.11.7.8)
Supplement: Supplement 1 [file tvst-11-7-8_s001.pdf]

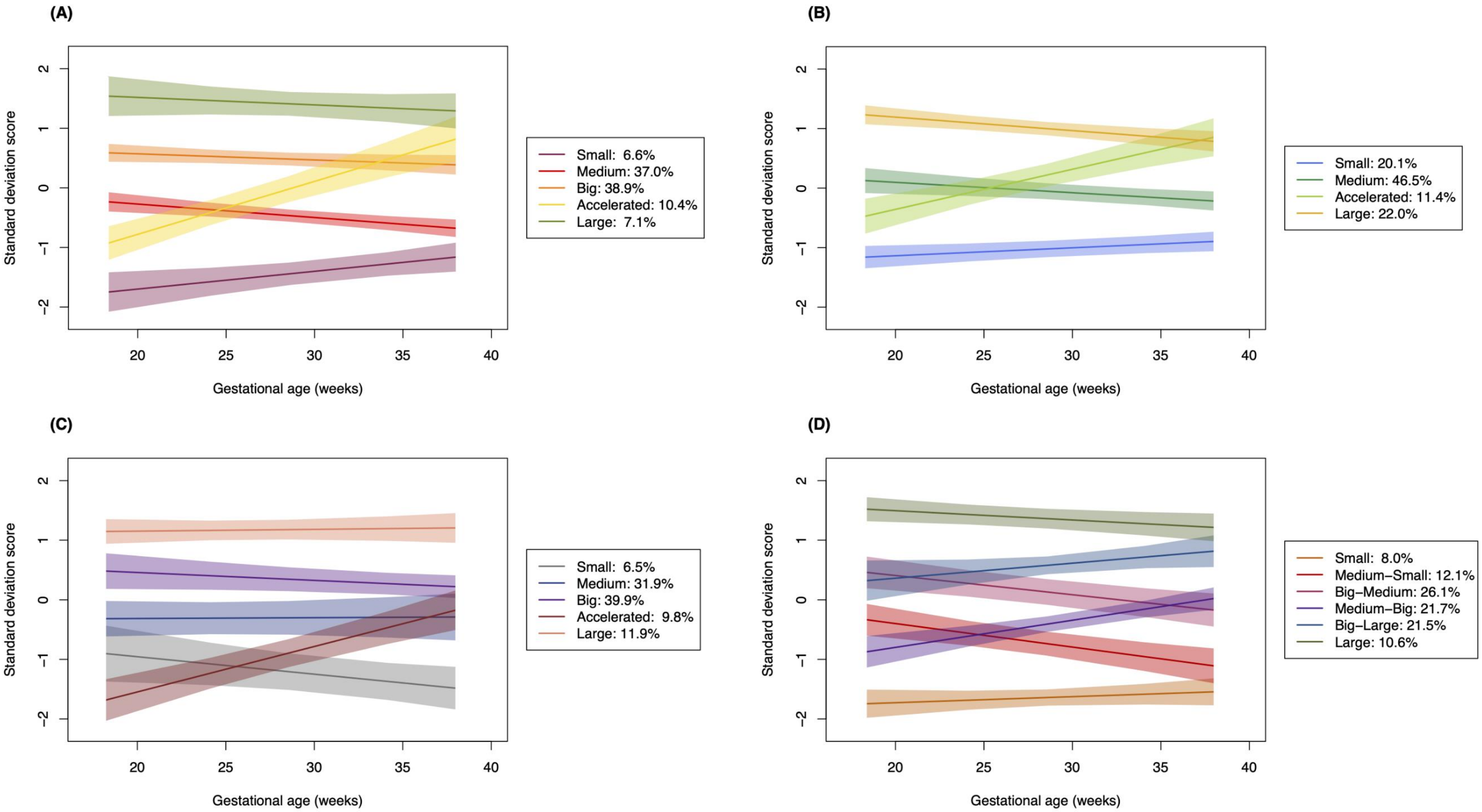
(A)
Standard deviation score
Gestational age (weeks)
Small: 6.6%
Medium: 37.0%
Big: 38.9%
Accelerated: 10.4%
Large: 7.1%
(B)
Standard deviation score
Gestational age (weeks)
Small: 20.1%
Medium: 46.5%
Accelerated: 11.4%
Large: 22.0%
(C)
Standard deviation score
Gestational age (weeks)
Small: 6.5%
Medium: 31.9%
Big: 39.9%
Accelerated: 9.8%
Large: 11.9%
(D)
Standard deviation score
Gestational age (weeks)
Small: 8.0%
Medium–Small: 12.1%
Big–Medium: 26.1%
Medium–Big: 21.7%
Big–Large: 21.5%
Large: 10.6%

Supplementary Figure S1: Plots of the trajectory groups (estimated mean trajectories with 95% confidence intervals) previously constructed by Dyer et al.[23]

(A) The five-group fetal head circumference trajectory model; (B) the four-group fetal abdominal circumference trajectory model; (C) the five-group fetal femur length trajectory model; and (D) the six-group estimated fetal weight trajectory model.

Percentages provided in legends represent size of each trajectory group as a proportion of the study population in the current analysis (n = 481).

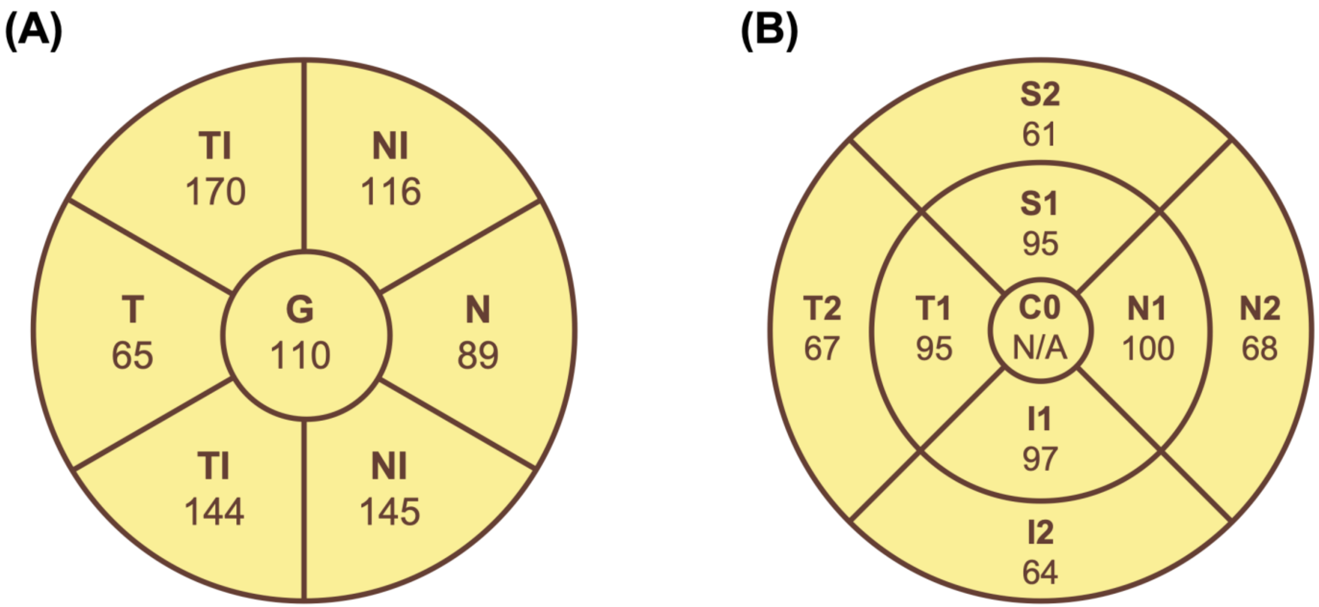

Supplementary Figure S2: Retinal nerve fiber layer and ganglion cell-inner plexiform layer thicknesses for a randomly selected study participant. (A) Retinal nerve fiber layer thicknesses (µm): global (G), temporal (T), superotemporal (TS), superonasal (NS), nasal (N), inferonasal (NI), inferotemporal (TI); (B) Sectoral ganglion cell-inner plexiform layer thicknesses (µm) with regions as defined by the Early Treatment Diabetic Retinopathy Study:[38] central macula (C0), inner temporal (T1), outer temporal (T2), inner superior (S1), outer superior (S2), inner nasal (N1), outer nasal (N2), inner inferior (I1), outer inferior (I2). Inner regions extend from a 0.5 to 1.5mm radius from the fovea and outer regions extend from a 1.5 to 3.0 mm radius from the fovea. N/A, not applicable given the ganglion cell-inner plexiform layer is absent at the central macula.
