# Supplementary material for: The Relationship Between Fetal Growth and Retinal Nerve Fiber Layer Thickness in a Cohort of Young Adults
Source: Transl Vis Sci Technol. 2022 Jul 12;11(7):8. doi: 10.1167/tvst.11.7.8 (PMC9287618; doi:10.1167/tvst.11.7.8)
Supplement: Supplement 2 [file tvst-11-7-8_s002.docx]

Supplementary Table S1: Maternal, neonatal and ophthalmic characteristics of participants in the trajectory groups of the four fetal growth trajectory models.

|  | Trajectory (Fetal Head Circumference Model) | | | | |  | Trajectory (Fetal Femur Length Model) | | | | |  |
| --- | --- | --- | --- | --- | --- | --- | --- | --- | --- | --- | --- | --- |
|  | Small | Medium | Big | Accelerated | Large |  | Small | Medium | Big | Accelerated | Large |  |
| n (%) | 28 (6.6%) | 157 (37.0%) | 165 (38.9%) | 44 (10.4%) | 30 (7.1%) |  | 31 (6.5%) | 153 (31.9%) | 191 (39.9%) | 47 (9.8%) | 57 (11.9%) |  |
|  | Pregnancy characteristics | | | | |  | Pregnancy characteristics | | | | |  |
| Fetal sex |  |  |  |  |  |  |  |  |  |  |  |  |
| Male | 14 (50.00%) | 74 (47.13%) | 84 (50.91%) | 25 (56.82%) | 16 (53.33%) |  | 19 (61.29%) | 74 (48.37%) | 99 (51.83%) | 19 (40.43%) | 28 (49.12%) |  |
| Female | 14 (50.00%) | 83 (52.87%) | 81 (49.09%) | 19 (43.18%) | 14 (46.67%) |  | 12 (38.71%) | 79 (51.63%) | 92 (48.17%) | 28 (59.57%) | 29 (50.88%) |  |
| Maternal smoking during pregnancy |  |  |  |  |  |  |  |  |  |  |  |  |
| Limited | 18 (64.29%) | 117 (74.52%) | 128 (77.58%) | 38 (86.36%) | 28 (93.33%) |  | 17 (54.84%) | 115 (75.16%) | 153 (80.10%) | 38 (80.85%) | 50 (87.72%) |  |
| Significant | 10 (35.71%) | 40 (25.48%) | 37 (22.42%) | 6 (13.64%) | 2 (6.67%) |  | 14 (45.16%) | 38 (24.84%) | 38 (19.90%) | 9 (19.15%) | 7 (12.28%) |  |
| Gestational age at birth (weeks) | 39.85 ± 1.53 | 39.74 ± 1.36 | 39.77 ± 1.44 | 40.11 ± 1.91 | 39.06 ± 1.60 |  | 39.32 ± 1.73 | 39.78 ± 1.58 | 39.60 ± 1.47 | 39.75 ± 1.84 | 39.18 ± 1.52 |  |
| Head circumference at birth (cm) | 33.23 ± 1.52 | 34.04 ± 1.35 | 35.13 ± 1.36 | 35.46 ± 1.36 | 35.33 ± 1.45 |  | 33.67 ± 1.97 | 34.46 ± 1.5 | 34.75 ± 1.44 | 34.34 ± 1.53 | 35.06 ± 1.45 |  |
| Birth weight (g) | 3062.14 ± 510.03 | 3252.01 ± 408.24 | 3571.36 ± 439.87 | 3583.64 ± 480.72 | 3637.67 ± 457.59 |  | 2986.61 ± 540.79 | 3341.97 ± 437.09 | 3456.99 ± 478.96 | 3273.40 ± 462.24 | 3669.04 ± 421.29 |  |
| Right axial length (mm) | 23.39 ± 0.78 | 23.43 ± 0.88 | 23.65 ± 0.89 | 23.57 ± 0.84 | 23.64 ± 0.76 |  | 23.33 ± 0.78 | 23.55 ± 0.96 | 23.52 ± 0.80 | 23.50 ± 0.76 | 23.83 ± 0.86 |  |
| Left axial length (mm) | 23.35 ± 0.81 | 23.42 ± 0.90 | 23.62 ± 0.89 | 23.55 ± 0.82 | 23.67 ± 0.82 |  | 23.27 ± 0.80 | 23.57 ± 1.01 | 23.50 ± 0.79 | 23.49 ± 0.83 | 23.82 ± 0.90 |  |
| Right IOP (mmHg) | 16.25 ± 4.57 | 15.74 ± 2.97 | 16.18 ± 3.17 | 15.55 ± 3.66 | 15.57 ± 3.63 |  | 15.00 ± 3.31 | 16.18 ± 3.28 | 15.72 ± 3.10 | 16.25 ± 4.24 | 15.83 ± 3.15 |  |
| Left IOP (mmHg) | 16.74 ± 4.60 | 15.33 ± 3.16 | 15.69 ± 3.14 | 15.37 ± 3.75 | 15.28 ± 4.12 |  | 15.06 ± 3.12 | 15.65 ± 3.38 | 15.49 ± 3.30 | 16.20 ± 4.30 | 15.32 ± 3.33 |  |

|  | Trajectory (Fetal Abdominal Circumference Model) | | | |  | Trajectory (Estimated Fetal Weight Model) | | | | | | |  |
| --- | --- | --- | --- | --- | --- | --- | --- | --- | --- | --- | --- | --- | --- |
|  | Small | Medium | Accelerated | Large |  | Small | | Medium-Small | Big-Medium | Medium-Big | Big-Large | Large |  |
| n (%) | 95 (20.1%) | 220 (46.5%) | 54 (11.4%) | 104 (22.0%) |  | 33 (8.0%) | | 50 (12.1%) | 108 (26.1%) | 90 (21.7%) | 89 (21.5%) | 44 (10.6%) |  |
|  | Pregnancy characteristics | | | | |  | Pregnancy characteristics | | | | | |  |
| Fetal sex |  |  |  |  |  |  | |  |  |  |  |  |  |
| Male | 50 (52.63%) | 110 (50.00%) | 22 (40.74%) | 53 (50.96%) |  | 15 (45.45%) | | 29 (58.00%) | 53 (49.07%) | 45 (50.00%) | 46 (51.69%) | 21 (47.73%) |  |
| Female | 45 (47.37%) | 110 (50.00%) | 32 (59.26%) | 51 (49.04%) |  | 18 (54.55%) | | 21 (42.00%) | 55 (50.93%) | 45 (50.00%) | 43 (48.31%) | 23 (52.27%) |  |
| Maternal smoking during pregnancy |  |  |  |  |  |  | |  |  |  |  |  |  |
| Limited | 71 (74.74%) | 164 (74.55%) | 49 (90.74%) | 85 (81.73%) |  | 21 (63.64%) | | 37 (74.00%) | 79 (73.15%) | 68 (75.56%) | 75 (84.27%) | 40 (90.91%) |  |
| Significant | 24 (25.26%) | 56 (25.45%) | 5 (9.26%) | 19 (18.27%) |  | 12 (36.36%) | | 13 (26.00%) | 29 (26.85%) | 22 (24.44%) | 14 (15.73%) | 4 (9.09%) |  |
| Gestational age at birth (weeks) | 39.58 ± 1.94 | 39.73 ± 1.46 | 39.84 ± 1.25 | 39.44 ± 1.41 |  | 39.61 ± 1.94 | | 39.88 ± 1.42 | 39.62 ± 1.48 | 40.05 ± 1.29 | 39.87 ± 1.32 | 39.38 ± 1.63 |  |
| Head circumference at birth (cm) | 33.87 ± 1.67 | 34.48 ± 1.41 | 35.32 ± 1.35 | 35.13 ± 1.44 |  | 33.22 ± 1.66 | | 33.72 ± 1.43 | 34.3 ± 1.31 | 34.91 ± 1.26 | 35.46 ± 1.31 | 35.48 ± 1.29 |  |
| Birth weight (g) | 3050.16 ± 513.78 | 3337.82 ± 378.48 | 3732.87 ± 339.61 | 3685.29 ± 464.78 |  | 2907.58 ± 485.47 | | 3063.20 ± 331.54 | 3347.69 ± 392.37 | 3443.28 ± 343.44 | 3752.08 ± 353.55 | 3736.25 ± 513.98 |  |
| Right axial length (mm) | 23.52 ± 0.97 | 23.59 ± 0.84 | 23.47 ± 0.75 | 23.53 ± 0.88 |  | 23.40 ± 0.95 | | 23.44 ± 0.92 | 23.46 ± 0.85 | 23.48 ± 0.79 | 23.73 ± 0.87 | 23.59 ± 0.80 |  |
| Left axial length (mm) | 23.54 ± 1.08 | 23.58 ± 0.83 | 23.41 ± 0.77 | 23.53 ± 0.89 |  | 23.35 ± 0.97 | | 23.44 ± 0.95 | 23.44 ± 0.84 | 23.47 ± 0.84 | 23.70 ± 0.86 | 23.60 ± 0.80 |  |
| Right IOP (mmHg) | 15.75 ± 3.30 | 15.60 ± 3.24 | 17.12 ± 3.58 | 16.09 ± 3.21 |  | 15.85 ± 3.57 | | 16.26 ± 3.39 | 15.28 ± 3.17 | 16.11 ± 3.47 | 16.19 ± 3.39 | 16.12 ± 2.75 |  |
| Left IOP (mmHg) | 15.75 ± 3.43 | 15.28 ± 3.36 | 16.31 ± 3.39 | 15.56 ± 3.47 |  | 15.94 ± 3.35 | | 16.22 ± 3.20 | 14.82 ± 3.14 | 15.61 ± 3.82 | 15.65 ± 3.35 | 15.88 ± 3.27 |  |

Data are summarized by number and percentage of the trajectory group for categorical variables or by mean and standard deviation for continuous variables.

Ophthalmic variables were measured at the Gen2-20 year follow-up.
