# Supplementary material for: The Relationship Between Fetal Growth and Retinal Nerve Fiber Layer Thickness in a Cohort of Young Adults
Source: Transl Vis Sci Technol. 2022 Jul 12;11(7):8. doi: 10.1167/tvst.11.7.8 (PMC9287618; doi:10.1167/tvst.11.7.8)
Supplement: Supplement 3 [file tvst-11-7-8_s003.docx]

Supplementary Table S2: Global retinal nerve fiber layer thicknesses of participants in the trajectory groups of the four fetal growth trajectory models.

|  | Trajectory (Fetal Head Circumference Model) | | | | | | | | | | | |  | |
| --- | --- | --- | --- | --- | --- | --- | --- | --- | --- | --- | --- | --- | --- | --- |
|  | Small | | Medium | | | Big | | Accelerated | | | Large | | *p*-value | |
| n (%) | 28 (6.6%) | | 157 (37.0%) | | | 165 (38.9%) | | 44 (10.4%) | | | 30 (7.1%) | | Unadjusted | Adjusted |
| Right Global RNFL (µm) | 99 (94, 105) | | 101 (94, 106) | | | 101 (94, 108) | | 99 (95, 106) | | | 105 (102, 110) | | 0.023* | 0.037* |
| Left Global RNFL (µm) | 103 (94, 106) | | 99 (95, 107) | | | 100 (94, 107) | | 100 (95, 105) | | | 105 (98, 109) | |  |  |
|  | Trajectory (Fetal Abdominal Circumference Model) | | | | | | | | | | | |  | |
|  | Small | | | Medium | | | Accelerated | | | Large | | | *p*-value | |
| n (%) | 95 (20.1%) | | | 220 (46.5%) | | | 54 (11.4%) | | | 104 (22.0%) | | | Unadjusted | Adjusted |
| Right Global RNFL (µm) | 99 (94, 105) | | | 101 (94, 107) | | | 103 (96, 105) | | | 103 (95, 110) | | | 0.25 | 0.44 |
| Left Global RNFL (µm) | 100 (94, 105) | | | 100 (94, 107) | | | 102 (96, 107) | | | 102 (95, 107) | | |  |  |
|  | Trajectory (Fetal Femur Length Model) | | | | | | | | | | | |  | |
|  | Small | | Medium | | | Big | | Accelerated | | | Large | | *p*-value | |
| n (%) | 31 (6.5%) | | 153 (31.9%) | | | 191 (39.9%) | | 47 (9.8%) | | | 57 (11.9%) | | Unadjusted | Adjusted |
| Right Global RNFL (µm) | 98 (94, 108) | | 101 (94, 106) | | | 102 (94, 109) | | 102 (97, 106) | | | 103 (95, 108) | | 0.26 | 0.52 |
| Left Global RNFL (µm) | 100 (94, 106) | | 100 (95, 106) | | | 102 (95, 108) | | 102 (93, 106) | | | 100 (95, 107) | |  |  |
|  | Trajectory (Estimated Fetal Weight Model) | | | | | | | | | | | |  | |
|  | Small | Medium-Small | | | Big-Medium | | Medium-Big | | Big-Large | | | Large | *p*-value | |
| n (%) | 33 (8.0%) | 50 (12.1%) | | | 108 (26.1%) | | 90 (21.7%) | | 89 (21.5%) | | | 44 (10.6%) | Unadjusted | Adjusted |
| Right Global RNFL (µm) | 100 (95, 107) | 101 (95, 106) | | | 101 (94, 106) | | 100 (94, 106) | | 104 (96, 111) | | | 103 (97, 109) | 0.16 | 0.36 |
| Left Global RNFL (µm) | 103 (94, 105) | 99 (95, 107) | | | 100 (95, 106) | | 99 (95, 107) | | 104 (95, 109) | | | 104 (98, 107) |  |  |

RNFL, retinal nerve fiber layer.

Data are summarized by median (interquartile range), presented to the nearest µm.

*p*-values have been calculated using generalized estimating equations in both unadjusted models and in models adjusted for gestational age at birth, maternal smoking during pregnancy, and axial length and intraocular pressure measured at the Gen2-20 year follow-up.

**^*^**Significant at *p* < 0.05.
