# Supplementary material for: The Relationship Between Fetal Growth and Retinal Nerve Fiber Layer Thickness in a Cohort of Young Adults
Source: Transl Vis Sci Technol. 2022 Jul 12;11(7):8. doi: 10.1167/tvst.11.7.8 (PMC9287618; doi:10.1167/tvst.11.7.8)
Supplement: Supplement 4 [file tvst-11-7-8_s004.docx]

Supplementary Table S3: Associations between global retinal nerve fiber layer thickness and fetal head circumference trajectory group membership, using the “small”, “medium”, “big” and “accelerated” groups as the reference.

|  | Reference Group – Small | | | | |
| --- | --- | --- | --- | --- | --- |
|  | Unadjusted | |  | Adjusted | |
| Trajectory Group | Estimate (95% CI) | *p*-value |  | Estimate (95% CI) | *p*-value |
| Medium | 0.26 (-2.06 to 2.58) | 0.83 |  | 0.28 (-1.96 to 2.52) | 0.81 |
| Big | 0.34 (-1.98 to 2.67) | 0.77 |  | 0.32 (-1.92 to 2.55) | 0.78 |
| Accelerated | 0.41 (-2.41 to 3.23) | 0.77 |  | -0.06 (-2.80 to 2.68) | 0.97 |
| Large | 4.04 (1.09 to 7.00) | 0.007* |  | 4.01 (0.99 to 7.04) | 0.009* |
|  | Reference Group – Medium | | | | |
|  | Unadjusted | |  | Adjusted | |
| Trajectory Group | Estimate (95% CI) | *p*-value |  | Estimate (95% CI) | *p*-value |
| Small | -0.26 (-2.58 to 2.06) | 0.83 |  | -0.28 (-2.52 to 1.96) | 0.81 |
| Big | 0.09 (-1.37 to 1.54) | 0.91 |  | 0.04 (-1.36 to 1.44) | 0.96 |
| Accelerated | 0.15 (-2.01 to 2.32) | 0.89 |  | -0.34 (-2.45 to 1.78) | 0.76 |
| Large | 3.79 (1.45 to 6.13) | 0.002* |  | 3.73 (1.27 to 6.20) | 0.003* |
|  | Reference Group – Big | | | | |
|  | Unadjusted | |  | Adjusted | |
| Trajectory Group | Estimate (95% CI) | *p*-value |  | Estimate (95% CI) | *p*-value |
| Small | -0.34 (-2.67 to 1.98) | 0.77 |  | -0.32 (-2.55 to 1.92) | 0.78 |
| Medium | -0.09 (-1.54 to 1.37) | 0.91 |  | -0.04 (-1.44 to 1.36) | 0.96 |
| Accelerated | 0.07 (-2.10 to 2.23) | 0.95 |  | -0.37 (-2.48 to 1.73) | 0.73 |
| Large | 3.70 (1.36 to 6.04) | 0.002* |  | 3.69 (1.25 to 6.14) | 0.003* |
|  | Reference Group – Accelerated | | | | |
|  | Unadjusted | |  | Adjusted | |
| Trajectory Group | Estimate (95% CI) | *p*-value |  | Estimate (95% CI) | *p*-value |
| Small | -0.41 (-3.23 to 2.41) | 0.77 |  | 0.06 (-2.68 to 2.80) | 0.97 |
| Medium | -0.15 (-2.32 to 2.01) | 0.89 |  | 0.34 (-1.78 to 2.45) | 0.76 |
| Big | -0.07 (-2.23 to 2.10) | 0.95 |  | 0.37 (-1.73 to 2.48) | 0.73 |
| Large | 3.63 (0.80 to 6.47) | 0.012* |  | 4.07 (1.13 to 7.00) | 0.007* |

Estimates and *p*-values have been calculated in generalized estimating equations in both unadjusted models, and in models adjusted for the covariates of gestational age at birth, exposure to maternal smoking during pregnancy, and intraocular pressure and axial length at the Gen2-20 year follow-up.

*Significant at *p* < 0.0125 (= 0.05/4 taking into account Bonferroni correction for four comparisons).
