# Supplementary material for: The Relationship Between Fetal Growth and Retinal Nerve Fiber Layer Thickness in a Cohort of Young Adults
Source: Transl Vis Sci Technol. 2022 Jul 12;11(7):8. doi: 10.1167/tvst.11.7.8 (PMC9287618; doi:10.1167/tvst.11.7.8)
Supplement: Supplement 5 [file tvst-11-7-8_s005.docx]

Supplementary Table S4: Sectoral retinal nerve fiber layer thicknesses, vertical cup-to-disc ratios and sectoral ganglion cell-inner plexiform layer thicknesses of participants in the fetal head circumference trajectory model groups.

|  |  | Trajectory (Fetal Head Circumference Model) | | | | |  | |
| --- | --- | --- | --- | --- | --- | --- | --- | --- |
|  |  | Small | Medium | Big | Accelerated | Large | *p*-value | |
| n (%) | | 28 (6.6%) | 157 (37.0%) | 165 (38.9%) | 44 (10.4%) | 30 (7.1%) | Unadjusted | Adjusted |
| Sectoral RNFL thickness (µm) | |  |  |  |  |  |  |  |
|  | Right Temporal | 72 (64, 78) | 72 (65, 79) | 71 (64, 78) | 73 (66, 82) | 77 (68, 83) | 0.039 | 0.034 |
|  | Left Temporal | 65 (61, 74) | 69 (62, 75) | 68 (60, 73) | 68 (62, 76) | 71 (65, 76) |  |  |
|  | Right Superotemporal | 142 (129, 150) | 143 (128, 154) | 140 (128, 150) | 138 (128, 149) | 144 (130, 158) | 0.28 | 0.12 |
|  | Left Superotemporal | 137 (118, 144) | 135 (123, 148) | 135 (121, 149) | 139 (126, 146) | 141 (124, 153) |  |  |
|  | Right Superonasal | 104 (95, 117) | 103 (90, 118) | 105 (92, 118) | 105 (91, 117) | 105 (90, 113) | 0.99 | 0.78 |
|  | Left Superonasal | 113 (106, 123) | 113 (99, 130) | 116 (100, 133) | 117 (101, 126) | 116 (95, 132) |  |  |
|  | Right Nasal | 77 (72, 87) | 79 (70, 88) | 81 (71, 90) | 81 (69, 90) | 82 (76, 93) | 0.020 | 0.002* |
|  | Left Nasal | 83 (75, 86) | 79 (69, 88) | 81 (72, 91) | 79 (71, 86) | 80 (72, 93) |  |  |
|  | Right Inferonasal | 104 (90, 120) | 108 (95, 123) | 113 (91, 129) | 106 (94, 120) | 118 (103, 136) | 0.28 | 0.21 |
|  | Left Inferonasal | 109 (90, 134) | 109 (97, 125) | 113 (93, 128) | 113 (97, 128) | 117 (102, 136) |  |  |
|  | Right Inferotemporal | 149 (132, 154) | 144 (130, 156) | 145 (133, 156) | 145 (138, 160) | 156 (137, 162) | 0.048 | 0.048 |
|  | Left Inferotemporal | 147 (136, 154) | 144 (131, 157) | 144 (131, 154) | 142 (131, 156) | 153 (145, 160) |  |  |
| Vertical cup-to-disc ratio | |  |  |  |  |  |  |  |
|  | Right VCDR | 0.33 (0.22, 0.40) | 0.36 (0.24, 0.42) | 0.33 (0.21, 0.46) | 0.35 (0.21, 0.45) | 0.37 (0.23, 0.47) | 0.71 | 0.13 |
|  | Left VCDR | 0.32 (0.25, 0.37) | 0.33 (0.25, 0.44) | 0.34 (0.25, 0.45) | 0.44 (0.34, 0.51) | 0.36 (0.27, 0.48) |  |  |
| Sectoral GCIPL thickness (µm) | |  |  |  |  |  |  |  |
|  | Right Inner Temporal | 92 (86, 96) | 94 (89, 99) | 93 (87, 98) | 94 (90, 98) | 94 (90, 100) | 0.41 | 0.65 |
|  | Left Inner Temporal | 93 (88, 97) | 95 (90, 99) | 94 (90, 99) | 94 (88, 99) | 97 (93, 103) |  |  |
|  | Right Outer Temporal | 65 (60, 70) | 69 (63, 72) | 69 (64, 72) | 70 (66, 74) | 74 (69, 77) | 0.0001* | 0.0002* |
|  | Left Outer Temporal | 65 (62, 70) | 67 (63, 71) | 67 (62, 70) | 69 (63, 73) | 69 (66, 73) |  |  |
|  | Right Inner Superior | 93 (89, 102) | 98 (94, 101) | 97 (93, 101) | 98 (95, 100) | 98 (94, 103) | 0.046 | 0.07 |
|  | Left Inner Superior | 93 (91, 100) | 98 (93, 102) | 97 (93, 101) | 98 (95, 101) | 98 (93, 104) |  |  |
|  | Right Outer Superior | 59 (56, 64) | 61 (58, 63) | 60 (57, 62) | 62 (56, 64) | 61 (59, 63) | 0.003* | 0.002* |
|  | Left Outer Superior | 59 (56, 64) | 61 (57, 64) | 60 (57, 64) | 62 (56, 65) | 63 (60, 66) |  |  |
|  | Right Inner Nasal | 97 (93, 104) | 101 (97, 106) | 99 (96, 104) | 101 (97, 104) | 102 (98, 108) | 0.23 | 0.21 |
|  | Left Inner Nasal | 99 (96, 100) | 101 (96, 106) | 100 (94, 104) | 100 (96, 105) | 102 (97, 106) |  |  |
|  | Right Outer Nasal | 61 (58, 65) | 65 (61, 67) | 64 (60, 67) | 65 (62, 69) | 65 (62, 68) | 0.011 | 0.005* |
|  | Left Outer Nasal | 63 (58, 69) | 66 (62, 70) | 66 (61, 69) | 65 (61, 68) | 68 (64, 71) |  |  |
|  | Right Inner Inferior | 93 (89, 98) | 98 (93, 101) | 97 (93, 102) | 97 (96, 100) | 101 (96, 103) | 0.097 | 0.11 |
|  | Left Inner Inferior | 95 (91, 99) | 97 (91, 101) | 97 (93, 101) | 97 (93, 102) | 99 (94, 105) |  |  |
|  | Right Outer Inferior | 56 (52, 59) | 57 (55, 61) | 57 (54, 61) | 59 (57, 62) | 60 (56, 63) | 0.021 | 0.009 |
|  | Left Outer Inferior | 56 (55, 61) | 58 (55, 61) | 58 (54, 61) | 58 (55, 62) | 59 (57, 63) |  |  |

VCDR, vertical cup-to-disc ratio; RNFL, retinal nerve fiber layer; GCIPL, ganglion cell-inner plexiform layer.

Data are summarized by median (interquartile range). Sectoral RNFL and GCIPL thicknesses are presented to the nearest µm.

*p*-values have been calculated using generalized estimating equations in both unadjusted models and in models adjusted for gestational age at birth, maternal smoking during pregnancy, and axial length and intraocular pressure measured at the Gen2-20 year follow-up. Both VCDR models were also corrected for the vertical Bruch’s membrane opening width.

**^*^**Significant at *p* < 0.05 for VCDR models; significant at *p* < 0.05/6 = 0.008 for sectoral RNFL models including a Bonferroni correction for six analyses; significant at *p* < 0.05/8 = 0.006 for sectoral GCIPL models including a Bonferroni correction for eight analyses.
